# MRI-Targeted Biopsies versus Systematic Transrectal Ultrasound Guided Biopsies for the Diagnosis of Localized Prostate Cancer in Biopsy Naïve Men

**DOI:** 10.1155/2015/571708

**Published:** 2015-01-27

**Authors:** Alexandre Peltier, Fouad Aoun, Marc Lemort, Félix Kwizera, Marianne Paesmans, Roland Van Velthoven

**Affiliations:** ^1^Department of Urology, Jules Bordet Institute, 1 Héger-Bordet Street, 1000 Brussels, Belgium; ^2^Department of Radiology, Jules Bordet Institute, 1 Héger-Bordet Street, 1000 Brussels, Belgium; ^3^Department of Data Management, Jules Bordet Institute, 1 Héger-Bordet Street, 1000 Brussels, Belgium

## Abstract

*Introduction*. To compare, in the same cohort of men, the detection of clinically significant disease in standard (STD) cores versus multiparametric magnetic resonance imaging (mpMRI) targeted (TAR) cores.* Material and Methods*. A prospective study was conducted on 129 biopsy naïve men with clinical suspicion of prostate cancer. These patients underwent prebiopsy mpMRI with STD systematic biopsies and TAR biopsies when lesions were found. The agreement between the TAR and the STD protocols was measured using Cohen's kappa coefficient.* Results*. Cancer detection rate of MRI-targeted biopsy was 62.7%. TAR protocol demonstrated higher detection rate of clinically significant disease compared to STD protocol. The proportion of cores positive for clinically significant cancer in TAR cores was 28.9% versus 9.8% for STD cores (*P* < 0.001). The proportion of men with clinically significant cancer and the proportion of men with Gleason score 7 were higher with the TAR protocol than with the STD protocol (*P* = 0.003; *P* = 0.0008, resp.).* Conclusion*. mpMRI improved clinically significant prostate cancer detection rate compared to STD protocol alone with less tissue sampling and higher Gleason score. Further development in imaging as well as multicentre studies using the START recommendation is needed to elucidate the role of mpMRI targeted biopsy in the management of prostate cancer.

## 1. Introduction

Prostate cancer is the second most common cancer among males and the sixth leading cause of cancer death worldwide with estimated 899 000 new cases and 258 000 new deaths in 2008 [1]. The worldwide prostate cancer burden is expected to grow to 1.7 million new cases and 499 000 new deaths by 2030 simply due to the growth and aging of the global population making the diagnosis and staging of this cancer of great medical and public interest [1]. Early detection of prostate cancer reduces cancer specific and overall mortality and improves men's future quality of life because it decreases the risk of being diagnosed and developing locally advanced or metastatic disease [2].

The current approach for diagnosis of prostate cancer which includes digital rectal examination (DRE), PSA, and transrectal ultrasound (TRUS) guided biopsy had several limitations. DRE is operator dependent and detects preferentially larger tumours located in the posterior peripheral zone of the gland [3]. PSA testing which is an unspecific prostate marker is also an imperfect strategy for the diagnosis of prostate cancer in its early phase [4]. TRUS guided biopsy had also its shortcomings. Due to sampling error >20% of prostate cancers that require definitive treatment are not detected in the first TRUS biopsy session [5]. Furthermore, prostate remains the only organ where biopsy is a blind uniform sampling technique due to poor visibility of cancer in 2D TRUS images and limited anatomical context to guide needles to suspicious locations in the 2D TRUS plane. Therefore, there is currently a need to improve early detection of prostate cancer.

Recent studies had suggested that multiparametric magnetic resonance imaging (mpMRI) is of interest for early detection of clinically significant prostate cancer when whole mount radical prostatectomy had been used as the reference standard [6–8]. With the increased recognition of the capabilities of mpMRI for detecting prostate cancer, attempts were made to incorporate mpMRI into routine prostate biopsies. MRI-targeted biopsies with software registration and elastic fusion of MRI images during real-time TRUS imaging are being used increasingly in surgical day to day practice [9, 10]. However, few clinical studies had reported on their role in biopsy naïve patients with no prior history of prostate cancer [11]. Thus, the purpose of our studies was to compare, in the same cohort of men, the detection of clinically significant disease in standard (STD) cores versus mpMRI targeted (TAR) cores. Our paper conforms well to the standards of reporting for MRI-targeted biopsy studies (START) recommendations [12].

## 2. Study Methodology

All men referred to Jules Bordet Institute between March 2012 and September 2013 with a clinical suspicion of prostate cancer due to an abnormal PSA and/or DRE were offered prebiopsy mpMRI with targeted biopsies when a lesion was found and standard systematic biopsies in all patients. Our cohort was unique because all patients included have not been studied in any previously published cohorts. Ethics approval in our institute covers the use of prospectively collected clinical information for clinical and prognostic studies.

## 3. Study Population

Over this period, 129 consecutive patients with no prior history of prostate cancer presented with a clinical suspicion of prostate cancer and 124 patients (96.1%) underwent mpMRI. Five patients (4.4%) were excluded because of MRI contraindications. All patients who underwent mpMRI accepted subsequent prostate biopsy. Fourteen patients with a previous negative biopsy were excluded from our cohort. In total, 110 patients (85.3%) made up the final cohort. Median age was 65.8 ± 7.1 years (IQR 59.5–70.7), median prebiopsy PSA was 6.9 ± 6.3 ng/mL (IQR 4.6–9.6), and median prostate volume was 44.0 ± 22.3 cc (IQR 35.0–59.0). Further patient characteristics are presented in Table 1.

## 4. Conduct and Reporting of the MRI

Patients underwent a mpMRI of the prostate on a 3 Tesla magnet Verio system (Siemens A.G., Erlangen, Germany) using an external multichannel phased array coil and an endorectal coil (Medrad Inc.). MR examination was conducted as follows:

- axial and coronal T2 weighted fast spin echo (TSE, ETL 13) sequences, 3 mm thick slices, TR/TE: 5210/155,
- axial diffusion weighted (DWI) high resolution sequence, readout-segmented EPI (RESOLVE), 3 mm thick slices, *b* = 50,1200 and ADC maps (with quantitative ADC evaluation), TR/TE: 4000/89,
- 3D CSI sequence, SE, 4.5 mm thick slices, TR/TE: 750/145, 4 averages, acquisition time: 10 min,
- axial T1 weighted 3D gradient echo sequence for DCE-MRI, 3.5 mm thick slices, 192^2^ matrix, TR/TE/FA: 5.6/2.1/25°, 5 sec time resolution, 58 time points, bolus injection of 0.1 mM/Kg Gd-DOTA,
- axial T1 weighted, fat-suppressed sequence for late postcontrast imaging of pelvis and node detection, 4 mm thick slices, TSE (ETL 3), TR/TE: 861/11, FOV 384 × 324.

Examinations were interpreted by a trained radiologist with 19 years' experience reading prostate MRI and a present rate of 130 exams/year. Reporting was done using a combination of parameters within priority order morphology on T2W images, DWI (ADC maps), DCE-MRI, CSI, and suspicious regions of interest (ROI) contoured on T2W axial slices for subsequent processing on biopsy US device. Interpretation criteria for the positivity of parameters were conformed to European Society of Urogenital Radiology (ESUR) recommendations in the Prostate Imaging Reporting and Data System (PI-RADS) version 1 document, but not formally expressed as PI-RADS score [13]. Each ROI was classified as malignant (modified after [14]) in a 3-score ordinal scale as follows:

- low suspicion (0 to 1 parameter positive),
- moderate suspicion (2 parameters positive, including DWI),
- high suspicion (3 to 4 parameters positive).

## 5. Conduct of the Biopsy

All patients were prescribed prophylactic antibiotics and received a fleet enema at least 12 hours prior to the procedure. The patients were placed in the left lateral decubitus position with bent knees. The transducer probe was covered with a condom and placed in the rectum. All biopsies were performed under local anesthesia (10 cc of 2% lidocaine) by a single surgeon (>100 procedures/year and >15 years of experience using TRUS guided biopsies) using 18-gauge automated spring loaded biopsy gun providing a 22 mm long tissue cores.

Ten patients with no lesions on mpMRI underwent 3D TRUS guided systematic biopsy according to the modified Gore protocol [15]. The details of the biopsy platform and description of the biopsy technique have been previously described [16]. Patients with lesions suspicious of cancer on mpMRI underwent MRI-targeted biopsy. The mpMRI with documented ROIs was imported from a CD-ROM and loaded into the image processing component of the Sonoace X8 ultrasound machine (Medison/koelis urostation), a 3D ultrasound guided prostate biopsy software registration system. Each biopsy was done by holding the end firing 3D TRUS probe (3D5-9EK) by the right hand of the operator without an external support, a process called freehand. Initially, a 3D referenced prostate image, named the panorama image, was constructed by integrating 3 sets of 3D TRUS volume data acquired from 3 angles to capture the entire prostate image. The stored MRI data set was manually aligned and automatically fused with the real-time US, overlaying the ROIs on the virtual 3D prostate model (nonrigid registration). A multipanel image was generated on the monitor showing real-time US, the corresponding axial and sagittal MR images, and the virtual 3D model. Standard systematic biopsy was performed first: 12 cores were taken from the peripheral zone and 2 to 4 cores from the transitional zone according to the volume of the prostate. Then, the same operator performed the transrectal biopsies of target lesions. During the standard protocol, the operator was aware of the location of the lesion on mpMRI but the target was not visible on the screen. Because MRI-targeted and standard biopsies were carried out in the same group of men, each biopsy core was potted and fixed separately in 10% formalin and its precise location was recorded. Each biopsy specimen, embedded in paraffin, was serially cut at 3 *μ*m intervals, and subsequently histochemically stained with a freshly made hematoxylin and eosin solution for the microscopy observation by the same senior uropathologist. For each patient, anatomoclinical parameters, including the location of each core, the number of total and positive cores, the length of positive cores, the percentage of neoplastic disease, and the Gleason score, were reported separately in order to determine the cancer yield of each approach and to assess both clinically significant and nonsignificant prostate cancer.

## 6. Statistical Analysis

For data collection, the suitable case report of the START checklist was used. The proportions of cores positive for clinically significant disease were compared using the chi-squared test. The proportions of men with clinically significant disease were compared using a McNemar chi-squared test for paired percentages. Median comparisons were made using Wilcoxon's test. The agreement between the TAR and the STD protocols was measured using Cohen's kappa coefficient. Cohen's kappa confidence interval was calculated by bootstrap. All of the tests were two-sided and performed with a 5% alpha risk.

## 7. Results

The results are presented following START recommendations. Overall, a total of 110 patients were included in this study and 175 lesions were found on mpMRI. The characteristics of the two protocols are summarized in Table 2. On average, 1.6 targets were identified per patient (range 0–4). One hundred patients had an MRI-targeted biopsy and the remaining had no suspicious targets. On mpMRI, 86/175 (49.2%) targets were classified as low suspicious lesions, 69/175 (39.4%) as equivocal suspicious lesions, and 20/175 (11.4%) as high suspicious lesions (Table 3). All targets were successfully sampled with at least one targeted core. On average, 16.0 biopsy cores were taken per patient (range 12–23), 14.6 standard cores per prostate (range 12–18), and 2.4 cores per target (range 1–4). Cross tabulation of the number of men in each Gleason score obtained for the targeted biopsies against the scores obtained for the standard biopsies is presented in Table 3. Prostate cancer detection rate of MRI-targeted biopsy was 62.7% with 41 patients having no cancer detected.

In the current study, definition of clinical significance was limited to histologic definitions only. The presence of any core with a Gleason score > 3 + 3 and/or a maximal cancer core length ≥ 6 mm defined clinically significant prostate cancer. The presence of >2 positive cores was sufficient to define clinically significant disease in the standard protocol only. The number of men diagnosed with clinically significant cancer was 50 with the TAR protocol versus 32 with the STD protocol. Cross tabulation of the number of men with no cancer and clinically significant and clinically nonsignificant cancer with the TAR protocol against cancers detected with the STD protocol is presented in Table 4. Agreement weighted Cohen's kappa coefficient between the two protocols for the diagnosis of clinically significant, clinically insignificant, or no cancer was 0.51 (95% CI: 0.37–0.65). Cross tabulation of the number of men in each Gleason score obtained for the TAR biopsies against the scores obtained for the STD biopsies is presented in Table 5. Agreement weighted Cohen's kappa coefficient between the two protocols to determine the Gleason score was 0.57 (95% 0.29–0.71%).

The proportion of cores positive for clinically significant cancer in TAR cores alone was 28.9% versus 9.8% for STD cores alone (*P* < 0.001). The proportion of men with clinically significant cancer was higher with the TAR protocol than with the STD protocol (*P* = 0.0008). The proportion of men with Gleason score 3 + 4 and 4 + 3 was higher with the TAR protocol than with the STD protocol (*P* = 0.003).

## 8. Discussion

In 2003, the standards for the reporting of diagnostic accuracy (STARD) initiative published recommendations for the full and transparent reporting of diagnostic studies, including the use of a flowchart describing outcomes for each study participant and a checklist of items to be described [17]. However, a systematic review to compare the accuracy of an MRI-targeted biopsy approach with standard transrectal biopsy for the detection of clinically significant disease had shown that the majority of studies of MRI-targeted biopsy were of low quality and did not conform well to these standards [18]. In addition, conflicting data were also reported in the published literature [19–22]. Thus, a consensus meeting composed primarily of urologists and radiologists with expertise in the field of MRI-targeted biopsy defined, in 2013, the standards required for the reporting of studies of MRI-targeted prostate biopsies [12]. Our study conforms well to these standards and adds to the evidence based research in the field because it allows comparison, data synthesis, and future meta-analysis with other studies. Furthermore, most studies did not compare the detection of clinically significant prostate cancer between MRI-targeted and standard approaches [23, 24] and only two studies, published recently, reports its findings according to the START recommendation [25, 26].

We had already reported in a previous study of biopsy naïve men higher detection rate using the 3D system of Medison/koelis urostation without MRI fusion compared to standard protocol (50% versus 33%, *P* < 0.05) [16]. Higher detection rate stems probably from a better distribution of prostate cores inside the prostate [16]. Afterwards, we had integrated MRI-targeted biopsy using the same system into our practice. Overall cancer detection rate of MRI-targeted biopsy was 62.7% in our cohort of biopsy naïve men with clinical suspicion of cancer. This is in line with previous studies evaluating the results of MRI-targeted biopsy (47%–64%) and higher than studies reporting on standard biopsy [18]. In addition, in our study, TAR protocol improved prostate cancer detection rate compared to STD protocol and demonstrated higher detection rate of clinically significant disease with fewer tissue samples removed from lesions. The yield of targeted biopsies was significantly higher than standard biopsies with a ratio of 28.9% of cancer on TAR cores versus 9.8% of cancer on STD cores. Our data are even more encouraging when comparing clinically significant prostate cancer detection rate between the 2 protocols; 20 patients with clinically significant disease on TAR cores had either no cancer or clinically insignificant disease on STD cores and 11 patients with no cancer on TAR protocol had clinically insignificant disease on standard cores. In contrast, one patient with a clinically significant disease on STD protocol has a negative biopsy from a low suspicious lesion on the TAR protocol, and 5 patients with no cancer on STD protocol had a clinically insignificant cancer on TAR protocol. Our findings are in line with a recent study with comparable design published by Mozer et al. [25]. However, in contrast to their studies, we did find a statistically significant difference of Gleason score between STD and TAR biopsy. In fact, we noted an upgrading from Gleason 6 on STD protocol to Gleason 7 on TAR protocol in 13 out of 110 patients with 3 patients having Gleason 4 + 3. A similar study found upgrading of Gleason score in 32% of cases with a large number of Gleason 4 + 3 [26]. In addition, no cancer was found in 75.6% of lesions classified as low suspicious lesions on mpMRI in our study. In contrast, we found a clinically significant cancer in 19/20 high suspicious lesions. Siddiqui et al. [27] demonstrated a good correlation between the level of radiologic suspicion on mpMRI and the D'Amico risk stratification. Moreover, low suspicious lesions on mpMRI were associated with either negative biopsies or clinically insignificant disease in another studies published by Yerram et al. [14]. In parallel, this diagnostic approach was found to support apparent patient benefits by limiting overdiagnosis and unnecessary treatments [26]. Recently, some authors reported the results of transperineal approach for biopsy following MRI/TRUS fusion [28]. They demonstrated a higher detection rate for the anterior zone. However, to date, no prospective comparison of the approach of biopsy after the MRI/TRUS fusion has been made.

Finally, our study had some limitations. First, incorporation bias is inherent to all studies comparing TAR protocol to STD protocol in the same patient. In our study, STD protocol was performed before the TAR protocol but the operator was aware of the location of lesions on mpMRI. Although the target was not visible on the screen when performing the standard protocol, knowing the location of lesions on mpMRI could have influence the conduct of the standard biopsy. Some groups have attempted to overcome this problem by having different operators take the TAR protocol and the STD protocol [26]. Second, we had used the START recommendations to define clinically insignificant disease, but a histological evaluation of the radical prostatectomy specimens would be more appropriate. Third, risk stratifications of suspicion of lesions were conformed to ESUR recommendations in the PIRADS version 1 document, but not formally expressed as PIRADS scores.

## 9. Conclusion

TAR protocol appeared to have improved clinically significant prostate cancer detection rate compared to STD protocol with less tissue sampling. TAR protocol upgraded and detected prostate cancer with higher Gleason score. Low suspicious lesions on mpMRI were associated with either negative biopsies or clinically insignificant disease whereas high suspicious lesions were associated with a clinically significant disease. However, limiting prostate biopsy to the TAR protocol will miss some clinically significant prostate cancer. Further development in imaging as well as multicentre studies using the START recommendation will help to elucidate the role of mpMRI targeted biopsy in the management of prostate cancer, in particular active surveillance and focal therapy.

## Figures and Tables

**Table 1:** General characteristics of the patients (*n* = 110).

	Mean	SD	Median	IQR	Range
Age (years)	65.1	7.1	65.8	59.5–70.7	48.0–79.2
Prostate volume (mL)	49.3	22.3	44.0	35.0–59.0	18.0–162.0
PSA (ng/mL)	8.4	6.3	6.9	4.6–9.6	0.7–40.0
Clinical stage					
T1 = 85					
T2 = 25					

SD: standard deviation; IQR: interquartile range.

**Table 2:** Characteristics of the two protocols (*n* = 110).

		STD protocol	TAR protocol
Median number of cores taken per prostate		14.6	2.4

No cancer		60	53
Gleason score (nb of men)	3 + 3	42	38
	3 + 4	7	15
	4 + 3	1	4

Clinically insignificant disease (nb)		18	6
Clinically significant disease (nb)		32	51

STD: standard protocol; TAR: targeted protocol; nb: number.

**Table 3:** Cross tabulation of degree of suspicions and Gleason scores obtained with the TAR protocol (*n* = 175 lesions).

		TAR protocol			
		No cancer	Gleason 6	Gleason 3 + 4	Gleason 4 + 3
Degree of suspicions	Low	65	20	1	0
	Equivocal	19	28	19	4
	High	1	5	12	3

**Table 4:** Cross tabulation of the number of men with clinically significant and clinically insignificant cancer detected.

		STD protocol		
		No cancer	Clinically insignificant disease	Clinically significant disease
TAR	No cancer	41	11	1
	Clinically insignificant disease	5	1	0
	Clinically significant disease	14	6	31

**Table 5:** Cross tabulation of Gleason scores obtained with the TAR protocol and the STD protocol.

		STD			
		No cancer	Gleason 6	Gleason 3 + 4	Gleason 4 + 3
TAR	No cancer	41	12	0	0
	Gleason 6	19	17	1	0
	Gleason 3 + 4	0	10	6	0
	Gleason 4 + 3	0	3	0	1
